# Early loss of astrocyte p38α MAPK reduces hippocampal neuroinflammation, enhances synaptic strength, and increases non-synaptic mitochondrial uncoupling in female mice during non-pathological aging

**DOI:** 10.1007/s11357-025-01948-4

**Published:** 2025-11-14

**Authors:** Caleb S. Bailey, John C. Gant, Hilaree N. Frazier, Josh M. Morganti, Meggie C. Coleman, Verda A. Davis, Hemendra J. Vekaria, Patrick G. Sullivan, Christopher M. Norris, Linda J. Van Eldik, David J. Braun

**Affiliations:** 1 Sanders-Brown Center On Aging, University of Kentucky, 789 S Limestone, Todd 547, Lexington, KY 40536 USA; 2 Department of Pharmacology and Nutritional Sciences, University of Kentucky, Lexington, KY 40536 USA; 3 Department of Neuroscience, University of Kentucky, Lexington, KY 40536 USA; 4 Spinal Cord and Brain Injury Research Center, University of Kentucky, Lexington, KY 40536 USA; 5 Department of Neurosurgery, Medical University of South Carolina, Charleston, USA

**Keywords:** Aging, Astrocyte, P38 MAPK, Neuroinflammation

## Abstract

The p38 mitogen-activated protein kinase has a well-characterized role in modulation of inflammatory processes throughout the body. In the central nervous system, p38 is primarily studied within neurons and microglia, most commonly in the context of neurological insult. The present study was designed to determine its function in astrocytes during non-pathological aging. We generated a conditional knockout model in which a tamoxifen-inducible *Aldh1l1* promoter drives *Cre recombinase* expression in mice with exon 1 of the p38α gene flanked by loxP sites. Knockout of astrocyte p38α was achieved via tamoxifen administration in young sexually mature mice at 3–4 months old. Animals were subsequently aged to 21–24 months prior to performing electrophysiological, immunohistochemical, and biochemical analyses. We found that early loss of astrocyte p38α was associated with a reduction in hippocampal neuroinflammation and concomitant enhancement of synaptic strength in aged female mice. In subsequent experiments in younger animals, the knockout reduced peripheral GFAP levels and increased non-synaptic mitochondrial uncoupling. These findings indicate that astrocyte p38α has wide-ranging effects on brain metabolism, inflammation, and synaptic function during the course of normal aging, including release of GFAP from the central nervous system to the periphery. Follow-up studies exploring the role of astrocyte p38α in various age-associated neuropathological contexts are warranted.

**Supplementary information:**

The online version contains supplementary material available at https://doi.org/10.1007/s11357-025-01948-4.

## Introduction

The p38 mitogen-activated protein kinase is broadly expressed and critical in both development and cellular stress responses. Of the four isoforms, the alpha isoform (p38α) has attracted particular attention due to its central role in inflammatory and metabolic processes across organ systems [1]. In the aging context, p38α is known to contribute to cellular senescence and associated functional changes, including within the brain. Most commonly studied in neurons and microglia, p38α is known to regulate synaptic plasticity in the former and pro-inflammatory responses in the latter [2]. Much less is currently understood about its role within astrocytes, but the existing literature indicates a surprisingly widespread impact on overall inflammatory status and neural function. For example, a study ablating astrocyte p38α prior to intracerebral injection of the bacterial endotoxin lipopolysaccharide [3] found that its loss reduced glial fibrillary acidic protein (GFAP) levels at the injection site, consistent with in vitro findings. Beyond this, the overall in vivo neuroinflammatory context was changed in complex ways. Some factors were elevated at early time points (peripheral immune cell infiltration), some at slightly later (microglial activation), and some elevated initially but reduced over time (Cox2 levels). The net effect seems to be selective enhancement of initial but not later neuroinflammatory changes, although the specific implications of this are unclear. Interestingly, a more recent study demonstrated that astrocyte p38α is integral to hippocampal synaptic and memory function. Astrocyte p38α was found to govern gliotransmitter release in response to intracellular calcium signals, in a manner contributing to long-term depression (LTD) [4]. Its loss from astrocytes not only abrogated LTD but also enhanced long-term memory in young adult mice. This accords with previous data showing that astrocyte endocannabinoid receptors, which signal through p38 [5], induce LTD and can impair working memory [6]. Together, this prior literature implicates astrocyte p38α as a central nexus in coordinating neuroinflammatory and neuronal responses.

Astrocytes undergo phenotypic changes during normal aging in a process involving p38 signaling [1, 7]. Given that both neuroinflammation and synaptic dysfunction are features of normal brain aging (8), we aimed to investigate the effect of astrocyte p38α suppression in the non-pathological aging context. Sexually mature adult mice (3–4 months of age) underwent selective genetic knockout (KO) of astrocyte p38α, followed by analysis of hippocampal neuroinflammation and synaptic function when the mice were old (21–24 months of age). We found that this early and specific KO was sufficient to reduce hippocampal neuroinflammation and enhance synaptic strength when the mice were old—but only in females. Intriguingly, this corresponded with earlier reduction in peripheral levels of the astrocyte activation marker GFAP and enhanced non-synaptic mitochondrial uncoupled respiration. These data indicate that inhibition of astrocyte p38α can re-program neural function during normal aging in a beneficial manner, in female mice.

## Methods

### Animal model

Mice homozygous for the p38α gene (*Mapk14*) with exon 1 flanked by loxP sites (floxed) (Jax #31129) (9, 10)were bred to possess a Tamoxifen-inducible *Cre recombinase* (*Cre*) under control of the astrocyte *Aldh1l1* promoter (Jax #29655) and a *Cre*-inducible red fluorescent protein (RFP) reporter (Jax #7909). Tamoxifen was administered by 4 weeks of Tamoxifen chow (Envigo Teklad TD.130860; 0.4 g/kg Tamoxifen Citrate USP) at 3–4 months of age in the primary aging study. For the subsequent explorations of peripheral GFAP levels and mitochondrial function, 5 consecutive daily intraperitoneal injections (75 mg/kg) of Tamoxifen (Sigma #T-5648) dissolved in corn oil (Sigma #C8267) were administered at 3–4 months of age to better control exposure level and minimize duration. The number of animals used per group and timing of endpoint collection after p38^KO^ at 3–4 months are detailed in the relevant results text and figure legends. All animal studies were performed in accordance with approved University of Kentucky IACUC protocols.

### Immunohistochemistry and biochemical assays

Animals were euthanized and perfused with PBS, with the left hemisphere dissected for biochemistry and the right taken for immunohistochemistry as previously published (9, 11). Briefly, the right hemisphere was immediately post-fixed in 4% paraformaldehyde for 24 h and cryopreserved in 30% sucrose at 4 °C for at least 5 days prior to sectioning. Coronal sections of 30-μm thickness were collected on a freezing microtome and stored in cryoprotectant until ready for use. To measure microglial morphology, tissue was blocked and incubated in 1:1000 anti-IBA1 primary antibody (Wako #019–19741) overnight at 4 °C, followed by 1:100 anti-rabbit biotinylated secondary antibody (Vector Labs, #BA-1000) and Vectastain Elite ABC kit (Vector Labs, #PK-6100). Stained sections were developed with 0.5% DAB (MilliporeSigma, #D5637) and counterstained in Mayer’s modified hematoxylin solution (Abcam, #ab220365) for visualization of nuclei. Slides were imaged on an Aperio ScanScope XT digital slide scanner using a 20 × magnification and analyzed in HALO v3.5 (Indica Labs) with the HALO Microglial Activation v1.4 algorithm that measures process thickness and length in proportion to cell body size to estimate ramified (resting) vs non-ramified (activated) morphology. Algorithm parameters (in microns): activation process thickness, 3.77; max microglia process radius, 15; maximum nuclear size, 10; minimum nuclear size, 3; microglial min cell body diameter, 3.44; microglia max fragmentation length, 0.4; fill nuclear holes, true. Hippocampal RFP was directly visualized with a DAPI counterstain in the mounting media (Biotium #23018), using a Nikon BioPipeline Slide scanner (Nikon, Tokyo, Japan) and 10 × Plan Apo lambda objective. Images were processed with denoise.ai and 2D deconvolution in NIS Elements v5.4 (Nikon) and imported into HALO imaging software for analysis of total area positivity and object count.

The hippocampus from the left hemisphere was dissected and flash frozen in liquid nitrogen prior to processing for cytokine measurement. Tissue was homogenized in PBS lysis buffer (1:20 w/v) containing 0.2× Halt Protease Inhibitor Cocktail with 0.5 mM EDTA (Pierce, #78442) and 1 mM PMSF. Homogenates were centrifuged at 12,000×*g* for 20 min, and supernatants collected for cytokine quantification by MesoScale Discovery ELISA assays according to manufacturer’s instructions (MSD #K152A0H). Ratio of phosphorylated p38 (Thr180/Tyr182) to total p38 (all 4 isoforms) was also measured by MSD kit (#K15112D). Plates were read by an MSD Quickplex SQ120 and analyzed by Discovery Workbench 4.0 software. Cytokines were normalized to protein loaded per sample, as determined by BCA assay, and are expressed as (pg/mL)/mg. For measurement of peripheral GFAP, NfL, and total Tau, the blood was collected in EDTA tubes (SAI Infusion Technologies #M500E) and centrifuged at 2000×*g* to separate plasma. Ten microliters of plasma sample was diluted with 15 uL of sample buffer and run on the MSD S-PLEX Neurology Panel (#K15639S) according to manufacturer instructions.

### Electrophysiology

Extracellular field excitatory post-synaptic potentials (EPSPs) were acquired from the hippocampal stratum radiatum CA1 region in 400-μm-thick hippocampal transverse slices, per our established methods (12). Slices were prepared in ice-cold ACSF (in mM) 128 NaCl; 1.25 KH2PO_4_; 10 glucose; 26 NaHCO_3_; 3 KCl; 2 CaCl_2_; and 2 MgCl_2_ without Ca^2+^ and transferred to a custom holding chamber with regular ACSF at 31.5 °C and gassed with 95% O_2-_5% CO_2_ until used. Individual slices were transferred for recording to a RC22 chamber (Warner, Co., Hamden, CT) and perfused with ACSF for recording. The oxygenated ACSF, delivered at 1.5–2 mL/min, was maintained at 32° ± 1 °C using an inline heater (TC2Bip, Cell Micro Controls, Norfolk, VA). Data were acquired and analyzed using pCLAMP 9, a 700 A amplifier, and a DigiData 1320 board for digitization (Molecular Devices, Union City, CA). Recording electrodes were pulled from glass capillaries (Fisher Scientific, Pittsburgh, PA) on a Sutter Instruments (Novato, CA) pipette puller, and had tip resistances of 3–8 MΩ when filled with ACSF. Voltage records were digitized at 2–20 kHz and low-pass-filtered at 1 kHz. Paired pulse and LTP induction/measurements were performed using a twisted bipolar stimulation electrode (0.0045-inch coated stainless steel; A-M Systems, Everett, WA) positioned in the Schaffer collaterals/commissural fibers of stratum radiatum. For all synaptic stimulation, pulse duration was a 100 μs bi-phasic current delivered by an A365 stimulus isolator (WPI, Sarasota, FL) controlled by Clampex 9 software (Molecular Devices). I/O curves were obtained using increasing current amplitudes of current ranging from 10–600 mA. Paired pulse recordings were obtained by delivering two 100 μs bi-phasic pulses (S1 and S2) at multiple inter-stimulus intervals (ISI) in milliseconds: 10, 20, 30, 40, 50, 75, 100, 125, and 150. Three measures were taken at each amplitude and ISI for the I/O and paired pulse and averaged for analysis. For LTP, a baseline measurement was taken for 30 min every 30 s at an intensity below population spike threshold. After a stable baseline was achieved, two 1 s 100 Hz pulses were then delivered with a 10-s interval. EPSPs were then elicited every 30 s at the same intensity used for LTP induction and recorded for 1 h. Analysis was completed offline using Clampfit9 (Molecular Devices) and statistically analyzed using ANOVA and Fisher’s LSD post-hoc comparisons in StatView (SAS Institute, Cary, NC). Synaptic strength, paired pulse facilitation, and LTP were calculated according to the formulas below. Data output from individual slices were averaged within-animal prior to overall group comparisons. *p*-values < 0.05 were considered significant. All results are expressed as mean ± SEM.

Synaptic strength

$$\left(\frac{\text{maximum EPSP slope}}{\text{maximum fiber volley amplitude}}\right)$$

LTP:

$$\left(\frac{post \text{LTP fEPSP slope}-basline\text{ fEPSP slope}}{post \text{LTP fEPSP slope}}\right)*100$$

Percent paired pulse facilitation:

$$\left(\frac{S1-S2}{S2}\right)*100$$

### Bulk hippocampal RNAseq

A portion of hippocampus was dissected (*n* = 4/group), lysed in RLT Plus Buffer, and RNA extracted by RNeasy Plus Mini Kit (Qiagen, cat. #74136). RIN scores and total RNA were measured with a Bioanalyzer at the University of Kentucky Health Care Genomics Core Laboratory. Samples were sent to Novogene for performance of transcriptome sequencing and analysis on Illumina platforms. Data cleaning was performed by in-house Novogene Perl scripts to remove reads with adapters, poly-N, and low quality. Cleaned raw reads were aligned with Hisat2 v2.0.5 (mm39 reference genome) and featureCounts v1.5.0-p3 used to count reads mapped to each gene. Differential expression analysis was performed with DESeq2Rpackage (1.20.0) and KEGG pathway enrichment analysis with clusterProfiler R package v3.8.1. Raw readcount was normalized for sequencing depth, *p*-values calculated with negative binomial distribution regression models, and FDR corrections by the Benjamini–Hochberg method. Raw dataset has been uploaded to the Gene Expression Omnibus database (GSE306683).

### Seahorse mitochondrial assay on isolated hippocampal mitochondria

Animals were asphyxiated with CO_2_, rapidly decapitated, and the brain rapidly removed. The hippocampus was dissected from left hemisphere and transferred to 2 mL ice-cold mitochondrial isolation buffer (IB: 215 mM mannitol, 75 mM sucrose, 0.1% BSA, 1 mM EGTA, and 20 mM HEPES at pH 7.2). The mitochondria were isolated as previously described (13). Briefly, tissue samples were Dounce-homogenized with 8–10 strokes, and centrifuged at 1300×*g* for 3 min. The supernatant was transferred to a fresh 2-mL tube and centrifuged at 13000×*g* for 10 min. The pellet was resuspended in 450 µL IB followed by exposing it to 1200 psi for 10 min in a N2 pressure chamber (Parr instruments #4635) to break open synaptosomes. The mitochondrial suspensions were transferred to 1.5-mL centrifuge tubes and spun at 13,000×*g* for 10 min. The pellets were resuspended in 50 µL IB and the protein estimation was carried out using BCA protein assay kit (Pierce, #23227). Seahorse XFe96 Flux Analyzer (Agilent Technologies) was used to obtain measures of mitochondrial bioenergetics in isolated mitochondria as previously published (13). The oxygen consumption rates (OCRs) were measured in the presence of mitochondrial substrates, inhibitors, and uncouplers of the electron transport chain to measure different states of respiration. The sensor cartridge of Extracellular Flux kit was hydrated according to the manufacturer’s instructions one day before assay. The day of the assay injection ports A to D of the sensor cartridge were loaded separately or in combinations of substrates, inhibitors, and uncouplers. The final concentrations of the modulators were 5 mM pyruvate, 2.5 mM malate, and 1 mM ADP (via Port A), 2.5 µM oligomycin A (via Port B), 4 µM FCCP (via Port C), and 1 µM rotenone and 10 mM of succinate (via Port D). These reagents were prepared in respiration buffer without bovine serum albumin (BSA) added (RB; 125 mM KCl, 20 mM HEPES, 2 mM MgCl_2_, and 2.5 mM KH_2_PO_4_, adjusted to pH 7.2). A total of 4 µg mitochondrial protein in a volume of 30 µL RB (with 0.1% BSA) were loaded per well. The assay plates were centrifuged at 3000 rpm for 4 min at 4 °C. Additional RB (with 0.1% BSA) was added to bring the starting volume to 175 µL. After calibration, the utility plate was replaced by the assay plate carrying mitochondria. OCRs were measured for states of mitochondrial respiration. State III (CI, complex 1) response in presence of 5 mM pyruvate, 2.5 mM malate and 1 mM ADP (Port A) was measured, followed by state IV response in presence of 2.5 μM oligomycin A (Port B). Sequentially, state V (CI, complex 1) and state V (CII, complex 2) rates were recorded in presence of 4 μM FCCP (Port C); 0.1 μM rotenone and 10 mM of succinate (Port D), respectively.

### Statistical analyses

All statistical analyses and figure generation, aside from RNAseq as noted in the section, were performed in GraphPad Prism v10.5.0 with standard error depicted in graphs. Two-way ANOVAs, Student’s *t*-tests, or Mann–Whitney *U* tests were used depending on the comparisons performed and assessment of normality, as specified in figure legends.

## Results

### Early loss of astrocyte p38α is maintained in aged animals with no effect on overall health status or mortality

**Fig. 1:**
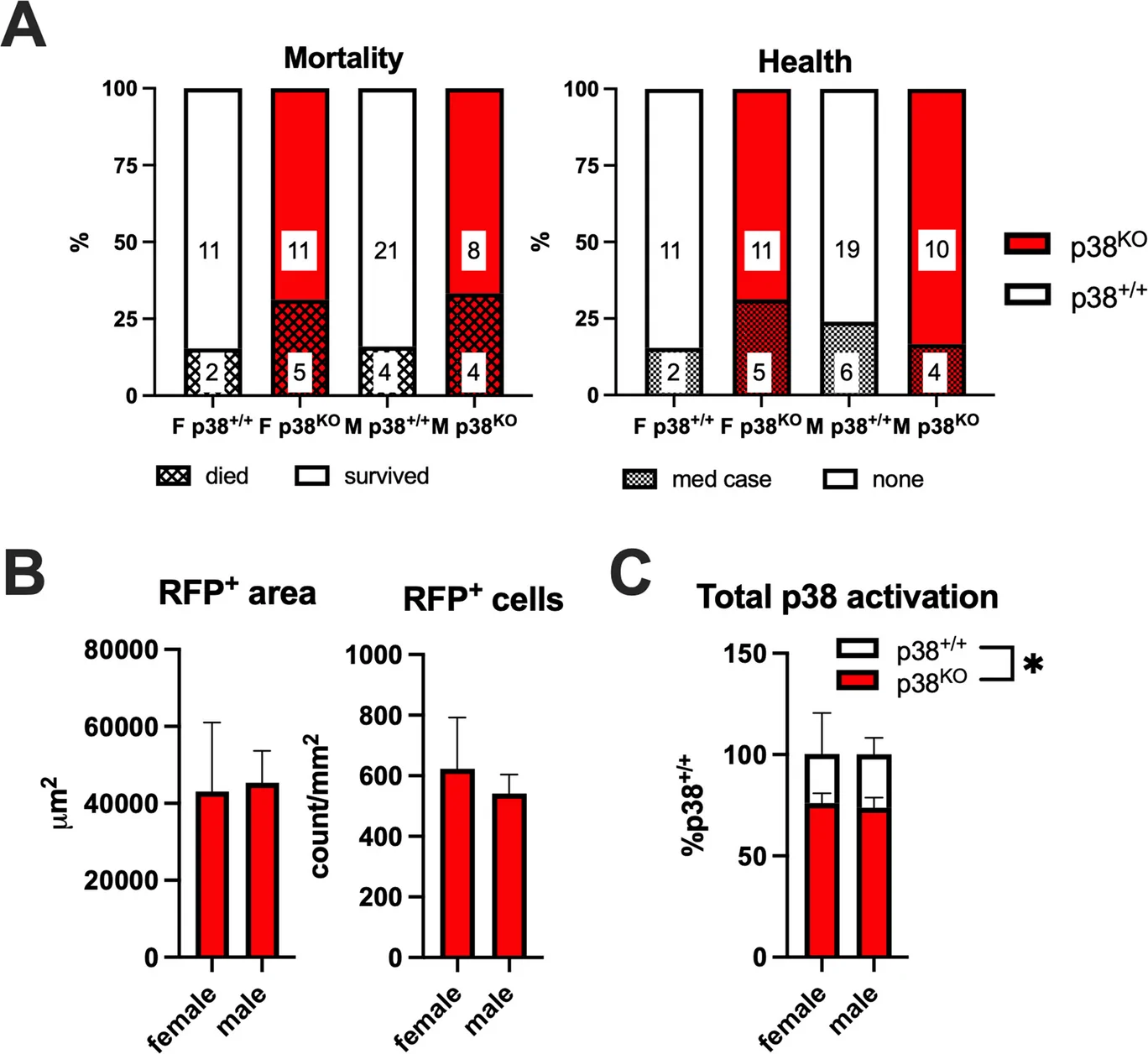
No differences in overall health or mortality resulting from astrocyte p38^KO^. **A** No significant differences in overall mortality or health (veterinarian-flagged medical cases) were observed after KO, up to ~ 22 months of age, *n* = 13–25/group, Fisher’s exact test. **B** No difference by sex in the total area positive or the number of RFP positive cells in the hippocampus, *n* = 3/group, Student’s *t*-test. **C** The ratio of phosphorylated to total p38 (activated p38) signal is expressed as a percentage of the p38^+/+^ group. The total activated p38 level is reduced by knockout of p38α, with no difference by sex, *n* = 4–10/group, *p < 0.05, two-way ANOVA

Initial construct-validation experiments demonstrated successful *Cre*-mediated recombination in ~ 85% of astrocytes, accompanied by an approximately 96% loss of astrocyte-specific p38α (Supplement 1). Subsequently, a group of p38α floxed mice, null for the *Aldh1l1-Cre recombinase* allele (p38^+/+^) or heterozygous carriers (p38^KO^), were administered tamoxifen at 3–4 months and allowed to age to ~ 21–24 months. Over this timespan, we did not observe significant alterations in health (Fisher’s exact test *p* = 0.80) nor mortality (Fisher’s exact test, *p* = 0.49) (Fig. 1A). We also found that the recombination was long-lasting as evidenced by robustly detectable RFP expression at endpoint collection (Fig. 1B). To determine whether compensatory increases in other p38 isoforms might occur, we measured total levels of p38 activation (phosphorylated p38/total p38, all isoforms) and found significantly reduced overall p38 activation in the p38α KO animals versus p38^+/+^ controls (Fig. 1C). Loss of this single isoform in a single cell type was sufficient to decrease overall p38 activation in brain by ~ 25%. Importantly, we did not observe significant sex differences in any of these metrics.

### Early loss of astrocyte p38α reduces neuroinflammation and enhances hippocampal synaptic strength in female but not male mice

**Fig. 2:**
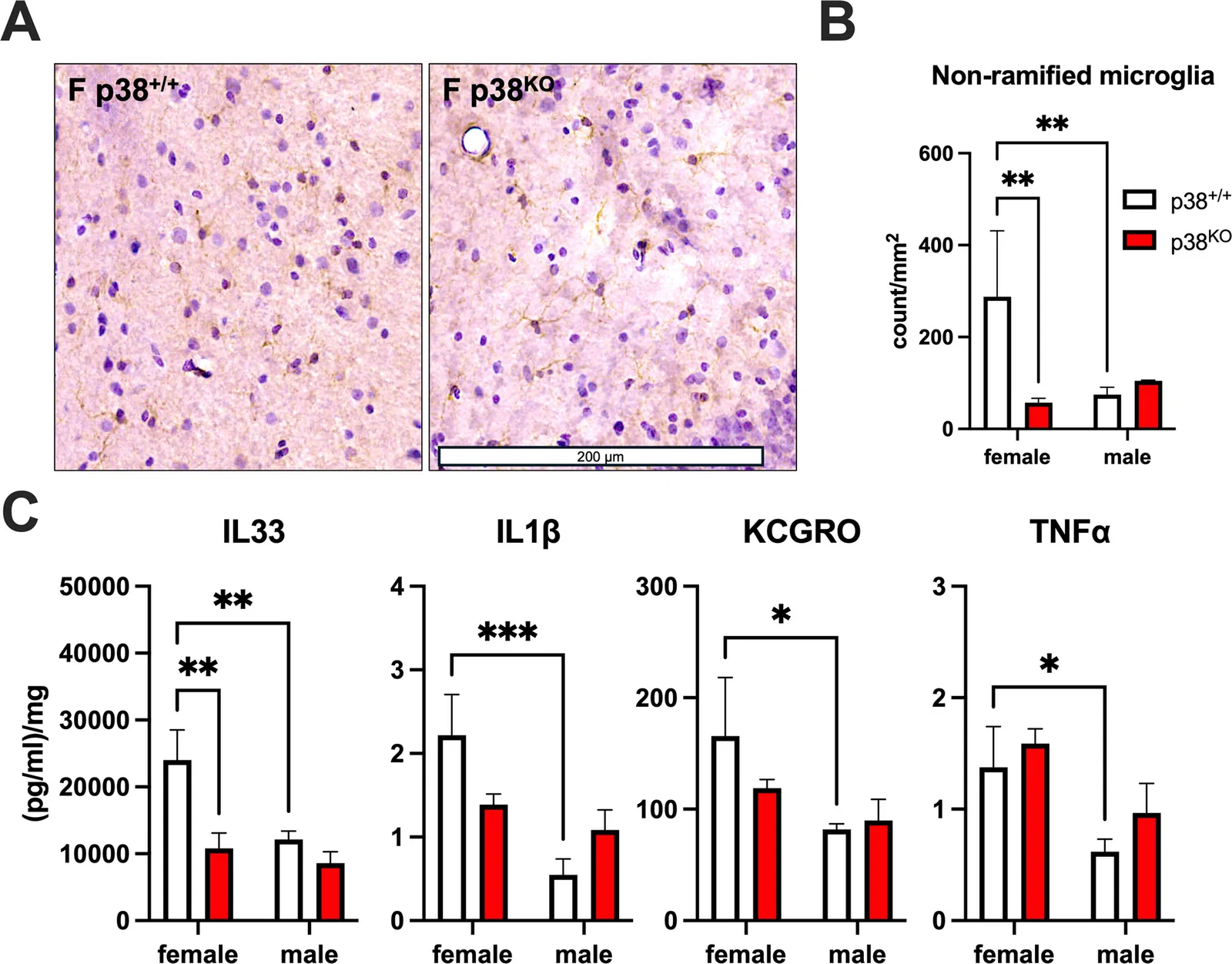
Astrocyte p38^KO^ reduces microglial activation and IL-33 levels in aged female mice. **A** Representative image (20×) with morphological analysis of IBA1 staining showing examples of microglia from p38^+/+^ or p38^KO^ females, with more ramified cell processes apparent in p38^KO^ animals. **B** Algorithmic quantification of morphologies showed significantly increased numbers of non-ramified microglia in females compared to males, an effect reduced by p38^KO^. **C** The loss of p38 was associated with a significant reduction in IL-33 in females, and a loss of sex differences in IL-1β, KCGRO, and TNFα. *n* = 3–10/group, *p < 0.05, **p < 0.01, ***p < 0.001 two-way ANOVA with Dunnett’s post-hocs

In accordance with reported sex differences in age-associated hippocampal neuroinflammation (14), we detected a morphological change in Iba1 + cells, as determined by changes in process length and thickness using the HALO microglial activation module (Fig. 2A, B). The Iba1 + cells had generally shorter and thicker processes in female mice as compared to males, classified by the algorithm as “activated” but which we refer to as “non-ramified.” These cells became more ramified in females after p38^KO^, in a manner consistent with a more “homeostatic” phenotype, we refer to as “ramified.” These morphological changes corresponded with changed cytokine levels within these same animals (Fig. 2C). Although the sex difference in IL1β, KCGRO, and TNFα were lost by the KO in females, only the reduction of IL33 reached significance in post-hoc tests. Levels of IL10, IFNγ, and IL6 were not different by sex or p38 status (not shown). The reduction of IL-33 in females with astrocyte p38^KO^ is especially notable, as it is secreted largely by astrocytes and plays a critical role in coordinating intercellular communication (15). Interestingly, there was no difference between groups in overall GFAP staining or total GFAP-positive cell count (two-way ANOVA, *F*(1,19) = 1.96, *p* = 0.18; two-way ANOVA, *F*(1,19) = 3.36, *p* = 0.08, respectively; data not shown) suggesting that, in the normal aging context, p38α may play a role in regulating the functional consequences of astrocyte activation rather than their activation state per se.

**Fig. 3:**
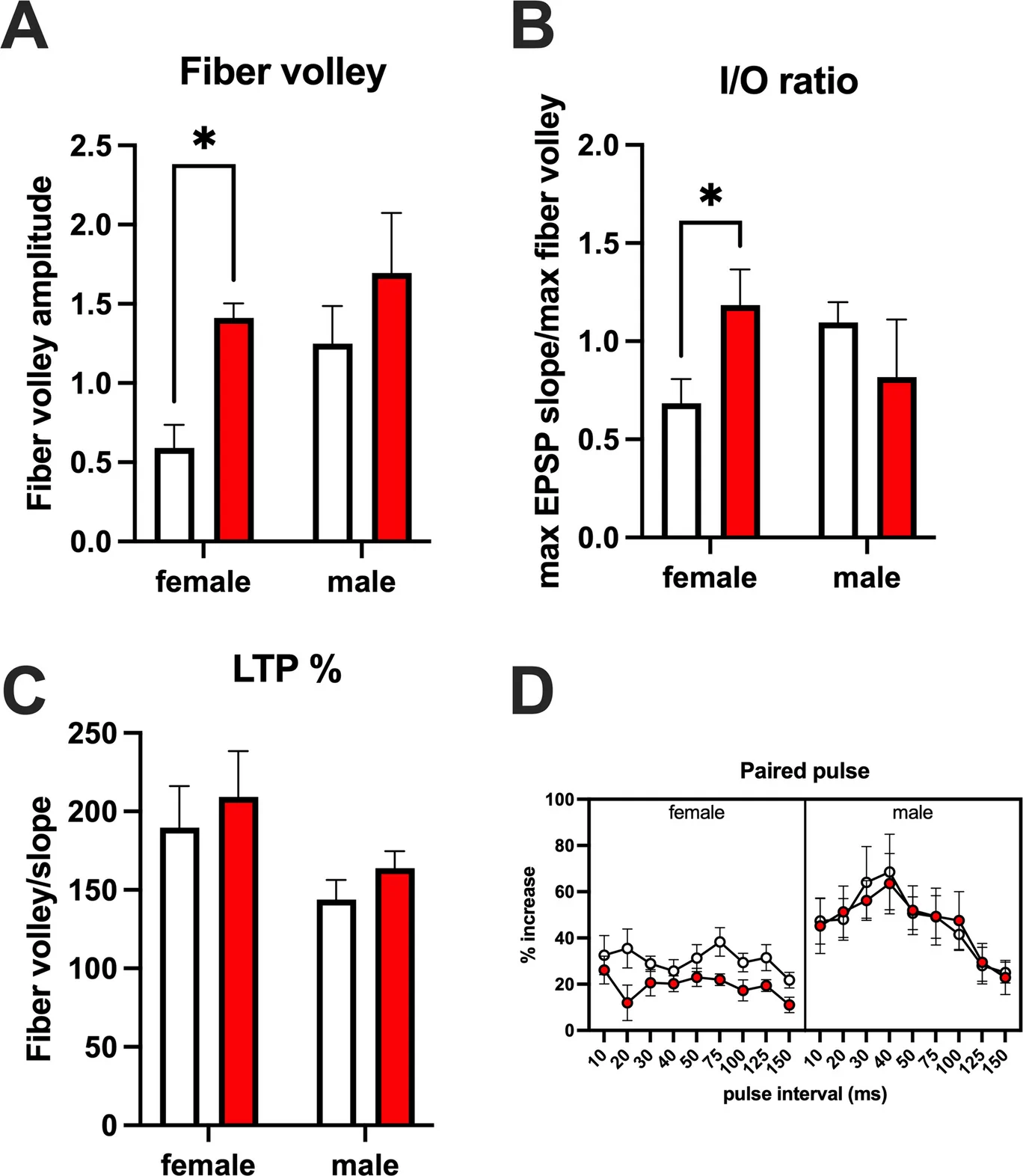
Astrocyte p38α^KO^ increases measures of neuronal excitability in the hippocampus of aged female mice. Field potentials were recorded from brain slices of hippocampus CA1 striatum radiatum in response to stimulation of CA3 Schaffer collaterals. Females with the KO had increased fiber volley amplitude (**A**) and I/O ratios (**B**) compared to those with intact p38. (**C)** p38 knockout did not affect LTP, *n* = 4–6 per group, *p <.05, **p <.01, two-way ANOVA with Fisher’s LSD. (**D**) There was no effect of p38^KO^ on paired pulse facilitation, but there was a significant main effect of sex (*p* < 0.01) by mixed-effects analysis

Alongside the neuroinflammatory changes, we detected divergences in measures of hippocampal synaptic function (Fig. 3). Fiber volley amplitude was enhanced in p38^KO^ female mice relative to female p38^+/+^, alongside an increase in overall synaptic strength reflected as the ratio of max EPSP slope/max fiber volley amplitude. No significant change was detected in long-term potentiation (LTP). Paired pulse analysis showed a significant main effect of sex, wherein potentiation is higher in aged males than females, but there was no significant effect of the knockout. Taken together, these findings indicate that astrocyte p38^KO^ in the aged female context enhances basal synaptic strength, likely involving both pre- and post-synaptic mechanisms.

**Fig. 4:**
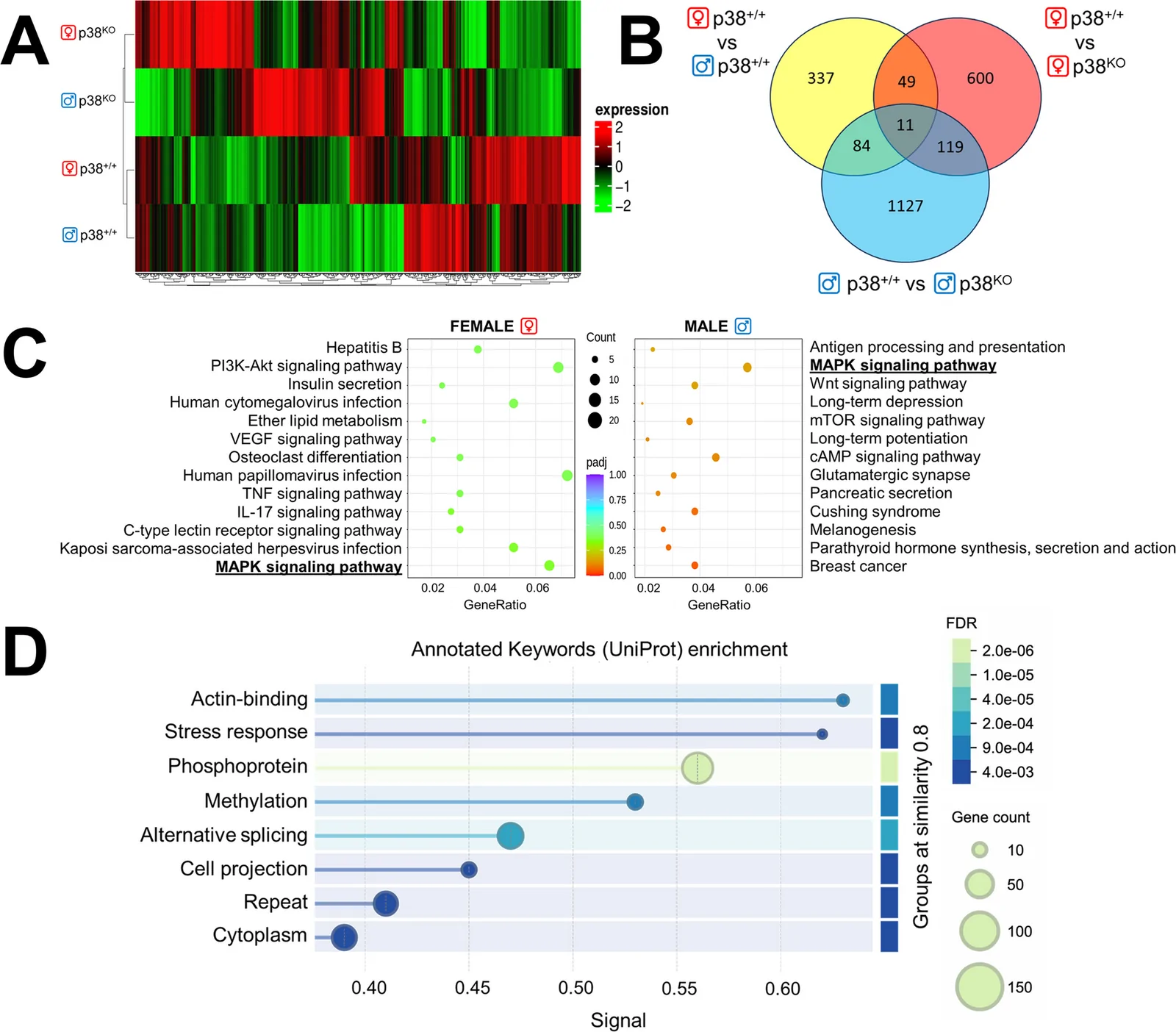
p38^KO^ produces sexually divergent changes in hippocampal gene expression in aged mice. Cluster analysis of groups by p38α status and sex (**A**). Of 1990 genes differentially expressed due to KO, 1211 were found uniquely in males (♂), 649 in females (♀), with only 130 in both (**B**). Of the overlapping genes, many are involved in synaptic signaling (*Homer1*, *Syn1*, *Syn2*, *Sv2b*, *Amph*, *Prkca*, *Prss12*, *Chrm3*, *Rab15*, *Grik4*) or control of neuronal excitability (*Kcnq2*, *Kcnq5*, *Kcnv1*). Of the most enriched KEGG pathways (**C**), only the MAPK signaling pathway appears in both sexes. (**D)** The 251 aging-associated hippocampal genes [16] that were also affected by p38^KO^ were analyzed for enrichment in STRING-DB. *n* = 3–4/group

These functional changes were further reflected by significant alterations in bulk hippocampal gene expression in a separate age-matched group of animals, as detected by RNAseq (Supplement 2 and GEO GSE306683). After FDR correction, 7 protein coding genes were found to be significantly different in female p38^KO^ versus p38^+/+^ mice, all reduced: *Cd109*, *Ccdc3*, *Nectin3*, *Trhde*, *Spock1*, *Mcmdc2*, and *Nkd1*. Seven protein coding genes were also found downregulated in males (*Hpgd*, *Lyrm7*, *Ccl28*, *Ublcp1*, *Dgkb*, *Ccnd1*, *Nectin1*), and 9 upregulated (*Srsf5*, *Akap8*, *Clk1*, *Clk4*, *Wsb1*, *Leng8*, *Col19a1*, *Depp1*, and *Coro6*). Notably, there is no overlap in these genes. Cluster analysis by group showed divergence by p38 status and sex in gene network changes (Fig. 4A). When DEGs with uncorrected *p* < 0.05 were examined, there was only modest overlap in the genes differentially expressed from p38^KO^ in the female versus the male mice, 130 in total (Fig. 4B). Gene expression changes from the KO included not only those highly expressed in mouse astrocytes (e.g., *Stat3*, *Ccdc3*, *Prodh*), but also in microglia (*Nfatc1*, *Serpinf2*), neurons (*Nxph3*, *Nkx2-1*), endothelia (*Adm*, *Folr1*), and oligodendrocytes (*Il12rb1*, *Adamtsl3*) (16). Pathway enrichment analysis further underscores the sex differences resulting from this intervention and the wide-ranging network effects of astrocyte p38 suppression (Fig. 4C), with the only enriched pathway shared between sexes being the MAPK signaling pathway.

We next compared this dataset with a list of 2086 age-associated DEGs (*p* < 0.05) reported in mouse hippocampus by Perez et al. (17). We found 251 DEGs from this list that are also altered by p38^KO^ in our aged mice, or about 12% (see Supplement 3). Enrichment analysis of these in STRING-DB (18) identified various hits, with actin-binding, stress response, and phosphoprotein highly enriched in line with known functions of p38 (Fig. 4D). In terms of sex, 194 of these 251 genes were found to be differentially expressed by p38^KO^ in males, 84 in females, with 27 that overlap between sexes. Stress response remained enriched in the female-only list, and actin-binding and phosphoprotein in the male-only list.

### Effects of astrocyte p38^KO^ in aged females correspond with an earlier reduction in peripheral GFAP levels and enhancement of mitochondrial uncoupled respiration

**Fig. 5:**
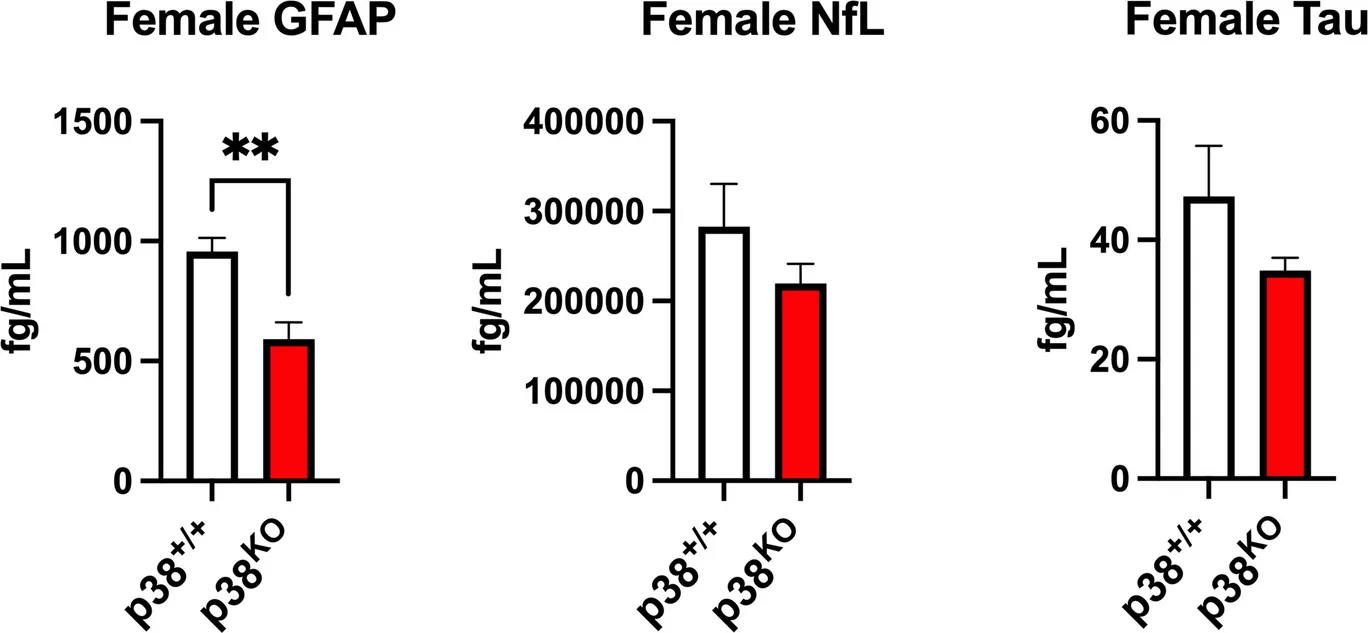
Peripheral levels of GFAP are reduced in female mice with astrocyte p38^KO^ by 8 months of age. Levels of plasma GFAP were found to be significantly reduced in 8-month-old females with p38^KO^ at 3–4 months of age. Reductions in plasma NfL and tau were non-significant. *n* = 6–7/group. **p < 0.01, Student’s *t*-test

To gain a better understanding of earlier changes that might underlie those apparent in the aged female animals, we carried out separate explorations of relevant endpoints. In particular, plasma levels of GFAP are considered a biomarker for astrocyte activation, known to increase both with age and in neuropathological contexts (19, 20). We therefore determined whether astrocyte p38α loss altered levels of peripheral GFAP. We measured plasma GFAP in a cohort of 8-month-old female mice that received Tamoxifen at 3–4 months of age. Loss of astrocyte p38α significantly reduced plasma GFAP levels with no effect on plasma neurofilament light chain (NfL), a generic biomarker of neuronal injury, nor total tau (Fig. 5). These data indicate loss of p38α may alter astrocyte function in a beneficial manner without neuronal perturbation.

**Fig. 6:**
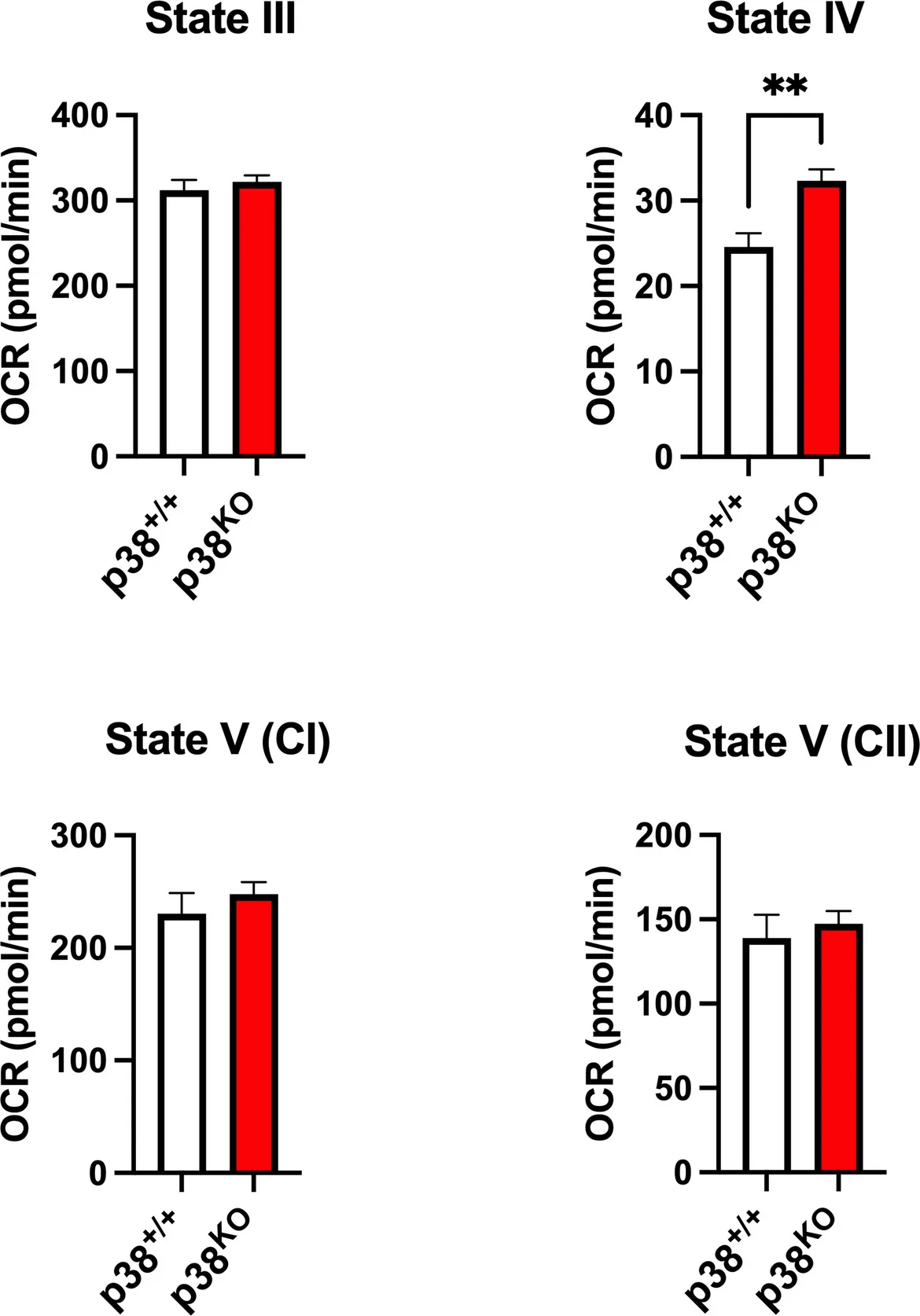
Astrocyte p38^KO^ promotes non-synaptic mitochondrial uncoupling by 12 months of age. Non-synaptic mitochondria were isolated from hippocampus of female mice at 12 months of age after p38^KO^ at 3–4 months of age. Loss of p38 significantly enhanced mitochondrial uncoupling (state IV), *n* = 7–8/group, **p < 0.01, Student’s *t*-test

To gain further insight, we next sought to determine whether brain metabolism might be implicated in the functional changes observed. Given that (a) p38 regulates mitochondrial function (21), (b) mitochondrial and metabolic abnormalities contribute to brain aging (22), and (c) our RNAseq dataset revealed that critical metabolic pathways are altered in aged female mice with p38^KO^ (Fig. 4), we decided to measure hippocampal non-synaptic mitochondrial function in females. Astrocyte p38 was knocked out at ~ 4 months of age with analysis of freshly isolated mitochondria when mice were 12 months, a period of known metabolic shift in females. The oxygen consumption rate (OCR) was measured by Seahorse assay as previously reported (23). No differences were observed in states III or V (complex I or complex II), but we found a significant increase in non-synaptic mitochondrial uncoupling (state IV) (Fig. 6). Taken together, we surmise that early astrocyte p38^KO^ in females is sufficient to alter the neural aging trajectory in a beneficial manner.

## Discussion

We found that astrocyte p38α activation contributes to overall hippocampal function and governs inflammation in the context of non-pathological aging in female mice. In particular, the loss of astrocyte p38α reduces broader neuroinflammation and enhances basal synaptic strength. Crucially, our findings do not appear to be primarily attributable to selective survival effects, nor differences in the extent of recombination or overall p38 suppression in male versus female mice. The sex differences are therefore due to some combination of sexual dimorphism in astrocyte p38α signaling or other divergences in the aged brain environment between males and females. Interestingly, sexually divergent effects of p38 KO from microglia have been reported in the neuroinflammatory context of experimental autoimmune encephalitis. Microglial p38 KO is protective in female but not male mice (24), due specifically to central and not peripheral effects (25) and dependent upon estrogen receptor signaling (26). While the present study was designed to determine the net effect of astrocyte p38α signaling in hippocampal aging—rather than comprehensively parse underlying mechanisms—our findings suggest several plausible and mutually inclusive avenues to pursue in future research, as discussed below.

Given the well-established role of pro-inflammatory cytokines in regulating synaptic function, the observed reduction in neuroinflammation and enhancement in synaptic strength are likely related. For example, IL-33 overproduction can cause deficits in hippocampal-dependent spatial memory via induction of microglial IL-1β (27, 28). This is straightforwardly in line with the observed concomitant reductions in microglial activation, IL-33, and IL-1β levels as a result of p38^KO^. However, there is no evidence that p38 directly controls IL-33 expression. The significant IL-33 elevation observed in aged females with intact p38α likely reflects a neuroprotective response to *other* aging-associated detrimental changes, such as oxidative stress-induced DNA damage (29). This interpretation better accords with reports that IL-33 improves hippocampal-dependent spatial memory (30, 31) and elevated levels are associated with reduced neuropathology in some contexts (32–35). Although not measured directly, a putative reduction of oxidative stress upstream of enhanced cytokine production is a particularly plausible explanation for several reasons: (1) oxidative stress is a core component of normal aging, (2) oxidative stress is a direct consequence of p38 overactivation, and (3) we observed an enhancement of mitochondrial uncoupling resulting from the loss of p38α. Uncoupling of oxidative phosphorylation reduces production of reactive oxygen species (36), and mild mitochondrial uncoupling can increase longevity across species (37). This literature might also explain a portion of the sex differences observed in our dataset. Male C57BL/6 J mice are known to have a longer lifespan than females, an effect at least partly attributable to elevated oxidative stress in females (38). Furthermore, brain metabolism shows sex differences, particularly after midlife when females show a greater drop-off in antioxidant capacity (39) and a shift from glucose utilization to alternative fuel sources such as lipids and ketone bodies (40). This shift, contributed to by so-called endocrinological aging, is hypothesized to underlie the particular susceptibility of women to develop Alzheimer’s disease. Although the role of p38 in metabolism is primarily studied in the periphery, the kinase is known to phosphorylate enzymes controlling glucose and lipid metabolism (41). While its precise metabolic effects in astrocytes are largely unknown, our finding of enhanced non-synaptic mitochondrial uncoupling indicate that p38α suppression may not only reduce production of reactive oxygen species but also increase the supply of lactate as a fuel source to neurons (42). While currently speculative, the direct quantification of oxidative stress burden and overall metabolic state as a function of astrocyte p38^KO^ will help to define the mechanism in future studies.

Aside from the neuroinflammatory and metabolic effects which may contribute to observed enhancement of synaptic strength, astrocyte p38α is also known to govern hippocampal gliotransmitter release in a manner that enhances synaptic depression and modulates memory function. Although we did not measure gliotransmitter release, this may represent a parallel mechanism contributing to the overall synaptic effects of p38^KO^ from astrocytes. Interestingly, long-lasting and contextually dependent alterations of neural function are programmed through modulation of p38 in microglia (25) and neurons (43), and the same seems true for astrocytes. Consistent with this idea, several earlier studies found similar neuroprotection in Alzheimer’s disease and related dementia (ADRD) models after astrocyte-specific inhibition of calcineurin/NFAT (44), JAK/STAT (45), and MyD88 (46), all of which interact with p38 to modulate neuroinflammatory signaling. Future studies incorporating functional behavioral endpoints are critical to interpret the net effect of astrocyte p38 signaling in overall neural function, and to pinpoint whether the sex effects lead to convergent or divergent behavioral performance.

We detected early reductions in peripheral GFAP levels without a corresponding change in GFAP levels in the aged hippocampus. GFAP is expressed in cells outside of the CNS, particularly Schwann cells of the peripheral nervous system, and certain cell types within the enteric system (47); furthermore, the *Aldh1l1* promoter is also expressed in some of these cell types (48), and the peripheral decrease in GFAP may therefore be due to systemic effects. It is also important to note that GFAP elevation does not represent the full spectrum of astrocyte activation (49), and we may be altering astrogliosis in some specific subset of cells that are not GFAP positive, or are located primarily outside of the hippocampus. If it is the case that the observed GFAP decrease is due to a reduction in efflux from the CNS, then our findings of reduced peripheral GFAP at a relatively young age indicate a role of p38 in basal GFAP production, its clearance, or both. In accordance with this interpretation, p38 activation has been implicated in both upregulation of GFAP (50) and its enhanced autophagy (51) in the context of various insults. Intriguingly, the brain-penetrant p38 inhibitor Neflamapimod reduces peripheral GFAP levels in patients with Lewy Body Dementia (52), an effect which the present study indicates may be due to direct action of the drug on astrocytes. Future work to better define these processes with more targeted CNS manipulation will help shed light on the implications of this particular finding within the context of therapeutic development.

Although a relatively small study, the convergent lines of evidence compiled herein point to astrocyte p38α as a crucial regulator of brain metabolism, neuroinflammation, and thereby overall function. Although we did not quantify non-astrocyte p38 levels, the *Aldh1l1* promoter is known to be highly specific to astrocytes within the brain, with some recombination in the subependymal zone (53), and it is therefore possible that other cell types were affected as well. Nonetheless, our data accords with the relatively sparse previous literature reporting neural effects of astrocyte-specific p38α loss in vivo, with regard to the broader neuroinflammatory [3] and synaptic changes [4]. As most studies to date have explored its role in microglia and neurons, and given clinical development of p38 modulators for treatment of aging-associated neurodegeneration (54–56), a better understanding of the role of p38α in astrocytes is highly topical. The question of whether the sex differences uncovered in this study reflect cell-autonomous or context-dependent divergences is particularly important to understand. That is, would beneficial effects be similarly apparent in male mice in the context of an insult? Studies to expand these findings and comprehensively define the astrocytic functional characteristics controlled by p38 signaling will help inform therapeutic targeting approaches across neuropathologies.

## Supplementary information

Below is the link to the electronic supplementary material.

ESM 1 (PNG 1.12 MB)

High Resolution Image (TIF 7.79 MB)

ESM 2 (XLSX 10.7 MB)

ESM 3 (XLSX 2.10 MB)

## Data Availability

The authors confirm that all data supporting the findings are available within the article, its supplementary files, and uploaded to GEO #GSE306683. Additional data are available upon reasonable request.
